# The British Journals: Surgery

**Published:** 1839-01

**Authors:** 


### SURGERY.

*On Lithotrity.* By W. Fergusson, f.r.c.s.e., Lecturer on Surgery, and one of the Surgeons of the Royal Infirmary of Edinburgh.

[This is a sensible paper, and is well deserving the attention of practical surgeons. The following extract will show that lithotrity is a less successful operation than is generally believed.]

I shall state briefly the particulars of certain cases which have occurred in my own practice, and the results of a considerable number more where I have had ample opportunities of making myself fully acquainted with the details.

Case i. A stout healthy man, about the middle period of life, underwent repeated operations with the lithotrite, all of which were attended with great pain; and, though at the end of several years no stone could be felt, he was in a worse condition than before coming under the surgeon's hands, in consequence of chronic disease of the bladder, apparently induced by the method of treatment.

Case ii. Mr. J. was operated on several times with very little suffering. After being fourteen days under treatment, only one small fragment could be detected, which, however, could not be readily grasped with the instrument: he went to the country, and shortly after passed a fragment, supposed to be the remainder of the stone. Not long after, symptoms of stone again came on, and, about eighteen months after the first operation, he submitted again to lithotrity, and the operation was performed with apparent success.

Case iii. J. W. submitted to lithotrity, and bore the first operation without much pain. In the subsequent attempts he suffered torment, but, notwithstanding, determined to persist, though it was frequently proposed to perform lithotomy. After repeated operations and great suffering, the bladder seems clear of all fragments; and ever since he has remained in good health.

Case iv. R. B. had suffered from stone in the bladder for forty years, firmly resolved never to submit to lithotomy. He felt anxious to undergo lithotrity, and put himself under my care for that purpose. I made an attempt to seize the stone without success; and, though the smallest possible degree of violence was used, still he suffered excruciating pain, which was followed by a feverish attack that did not leave him for several months, and since then he remained in a very exhausted condition. After his health was somewhat restored, he at last submitted to the operation of lithotomy, and I removed a large mulberry stone, weighing nearly five ounces. He made a rapid recovery.

Case v. The Rev. M. A. subjected himself to lithotrity, but suffered so much after the first attempt that it was not considered advisable to proceed. Twelve months afterwards he put himself under my charge, and I removed a small stone by the lateral operation. He made a slow recovery; but, like the patient whose case is last referred to, declared that, in the event of being again the subject of this disease, he would rather submit a second time to lithotomy than to lithotrity.

CASE VI. Mr. M., much against my advice, submitted to lithotrity: the operation, though performed with the least possible violence, was followed with excessive irritation and pain in the bladder, which continued for the next twelve hours, but was subdued by powerful opiates. In thirty-six hours the pain returned, and continued without intermission until death, which happened four days after the operation was done. On dissection, the fragments of a large stone were found in the bladder, and a small one untouched. The prostate gland was enlarged, more particularly the middle lobe. The bladder was sacculated, but there was no trace of any injury having been inflicted on it during the operation. Both kidneys were much diseased in this case, and the psoas muscle on the left side seemed converted into a soft mass resembling coagulated blood.

CASE VII. Mr. C. was severely afflicted with symptoms of stone. He consulted me as to the propriety of lithotrity, but I strongly dissuaded him from it. Shortly afterwards, however, he put himself under the care of a professed lithotritist, who broke the stone on one occasion, but could never induce him to submit to a second operation. He has since then suffered much, and is now in a state of great misery; being, no doubt, in a more deplorable state than before the stone gas broken into fragments.

Many other cases have come under my own knowledge or observation, with which, however, I was not so particularly connected as those referred to above. I shall therefore only mention them in the following list, which will show the result of my experience and knowledge in cases of lithotrity, which have not been described in any accounts on the subject as yet published.

Out of eighteen cases in which lithotrity was performed, six have been cured, seven not cured, and five have died.

In one of the number of cured there are strong reasons to suspect a return of the disease; in another, though no stone can be felt, the patient has suffered almost as much since he was operated on (in consequence of disease of the bladder induced thereby) as he did previous to coming under the surgeon's care. Indeed, in two of these cases only can the operation be said to have been attended with that happy success which has been so generally claimed for lithotrity.

Of the seven not cured, four afterwards underwent lithotomy, of whom one died and three recovered.

This statement, which differs so very widely from those made by the professed advocates of lithotrity, appears somewhat startling, particularly to those individuals who have had no personal experience on the subject, and who may have given implicit reliance to the statement of M. Civiale, that, out of 244 cases, 236 have been operated on with success; and though it is probable that other surgeons in this country might show their experience in lithotrity to be more favorable than some has been, I feel satisfied that, in Scotland at least, the average of success will not stand higher than I have stated above.

*Edinburgh Med. and Surg. Journal. October* 1, 1838.

---

### *On a new Method of treating Burns.* By EDWARD GREENHOW, M.D., North Shields.

[THIS is a very important practical communication; and bears likewise, as we shall have occasion to show in our review of Dr. Macartney's recent work, on an important principle in the history of inflammation, which has received, for the first time, due attention from that distinguished teacher.]

It is somewhat more than twenty-five years since I was consulted for a boy, who had fallen with both his arms into a kettle of boiling pitch: the agony he suffered at first was extreme, but, as the pitch became cool, so did the pain abate.

Upon examination, I found his hands thickly coated with pitch, which also had found its way up the jacket-sleeves, and probably also through the texture of the cloth: at all events, the sleeves from the wrists almost to the shoulders were firmly glued to the arms in a solid compact mass. The pitch on the hands, after much trouble, was got off by the free use of sp. terebinthinæ, and they were dressed with ung. resin. flav. cum ol. terebinth.; but with the arms I could do nothing. After vainly attempting to dissolve the hardened mass, I abandoned it in despair, by no means easy as to the result of leaving it alone: however, he made little complaint of the arms, and in the meantime the hands suppurated copiously, sloughs separated, granulations rose, and skin began to form in various points. It was now three weeks since the accident, and there had been no appearance of discharge from the arms, no offensive smell; nothing, in fact, to indicate that any process was going on. At the end of that period, however, I was delighted to find the sleeves of the jacket begin to loosen, and detach themselves from the arms; and in two or three days more I was enabled to rip them up and remove them altogether, when, to my surprise, I found the arms perfectly healed, and covered with a new skin; whereas the hands were not entirely healed until a month afterwards. On closely examining the jacket-sleeves, there was found adhering to them a substance resembling thin leather, which I could not doubt was a slough which had separated from the arms: and this had taken place, and the whole healing process had been accomplished without the intervention of suppuration.

This case made a great impression upon my mind, and I endeavoured to devise plans for imitating the coating of pitch, which had so wonderfully healed the burn which it had itself occasioned. In talking the case over with a friend in Newcastle, who was largely engaged in attendance upon collieries, in which cases of burns were of frequent occurrence, we agreed that, on the first occasion that presented itself, instead of dressing, as we had been accustomed, with ung. resin. flav. cum ol. terebinth. spread upon lint, to use the same ointment, melted over the fire, and applied with a brush or bunch of feathers, so as to form a complete coating over the burnt surface. It was not long before an opportunity presented itself of trying this plan upon an extensive scale; for, not many days afterwards, the friend above mentioned was summoned to a colliery where an explosion had taken place, and twenty-nine individuals, men and boys, were brought up, all of them more or less burnt, and many of them extensively and severely: all the brushes and feathers of the village were immediately called into requisition, and with the assistance of the relatives of the sufferers in a very short space of time they were all thickly coated with the ointment: they uniformly expressed themselves as much relieved, and they experienced none of the rigors so distressing after a burn, and the certain prelude to suppuration. The assistants were strictly enjoined to preserve the perfect integrity of the coating, by renewing it as often as it became necessary; and the men themselves were cautioned to move as little as possible, that they might avoid rubbing off the coating. It is unnecessary to dwell long upon these cases: they all recovered; many of them without the slightest suppuration taking place, and others having it take place only in a very partial degree; and, although the faces of some of them were severely burnt, no suppuration took place in any one of them; and it was only in places where friction could not altogether be avoided that suppuration took place at all; and on those parts where sloughs necessarily formed, in consequence of the depth of the injury, the sloughs peeled off like pieces of shrivelled leather, as the surface skinned beneath them.

The result of these cases was most satisfactory, establishing not only the possibility of healing burns without suppuration, but also that this was accomplished in a much shorter period than could have been effected by any other mode of treatment; and also that the constitutional irritation bore no comparison to what it would have done had there been a larger suppurating surface: in point of fact, many of these men were burned so extensively that, had suppuration taken place, the probability is that the system would have sustained a shock which would have proved fatal.

Since the period at which this occurred, I have pursued the same plan in the treatment of burns, and every succeeding year has served but the more to convince me of the advantages it possesses; and the friend I before alluded to never employed any other mode of treatment up to the end of his life.

[In a subsequent Number of the Gazette (November 3d,) a mode of treatment, considered by the proposer as analogous, is described by Mr. Leach as having been employed by him in sixty-five cases, with considerable success: this consists in the use of *treacle* spread, cold, upon fine calico.]

*Med. Gazette. October* 13, 1838.

---

*Vesico-vaginal Fistula cured by a new Mode of Operating.* By J. M. COLEY, Esq., Surgeon to the Bridgnorth Infirmary.

[THIS case is extremely creditable to Mr. Coley, and the modification in the mode of operating proposed by him is worthy of attention; although we cannot but think the very successful result obtained in his case must have been as much owing to some happy disposition of the patient's constitution as to the peculiarity of the operation. The following is Mr. C.'s statement of the advantages of his operation, and the history of the case in which it proved so successful.]

The operation is free from the objections connected with Mr. Earle's plan, inasmuch as it requires no previous dilatation of the urethra, and it admits of excision of the callous edges in the most favorable direction, i. e. upwards upon the instrument in the urinary passage; only one operation, if properly executed, is necessary; and I believe that the patient may be allowed to lie either on the side or back, instead of the abdomen, as insisted upon by all who have written on the subject.

CASE. 1835, July 8. A married woman, ætat. 40, had been labouring under vesico-vaginal fistula during four months. The urine was constantly passing through an opening between the bladder and vagina, three fourths of an inch long, nearly transverse, and situated just beyond the neck of the bladder.

The patient being placed on a high table, in the same position as for lithotomy, I introduced into the bladder a wood sound, one third of an inch in diameter, and the vagina being expanded laterally by assistants, the fistulous opening was brought into view by means of the sound, which, being depressed at its farther extremity, readily brought downwards and forwards the bladder and urethra. I then, with a scalpel, cut down upon the sound, through the vesico-vaginal structure, on each side the fissure, and without loss of time passed two ligatures, by means of a small curved needle, fixed in a suitable direction in a pair of forceps of a peculiar construction, the handles of which were fastened with a sliding ring. As soon as the needles were carried through the opening, from side to side, they were extracted by an assistant, with a pair of pocket forceps; and the divided parts being accurately brought into contact, I tied the ligatures with the fingers alone, without any delay or difficulty. The sound was now withdrawn, and a silver catheter introduced; when we had the satisfaction of seeing the urine flow through it, while none escaped by the vagina. The patient was next placed in bed, on the side opposite that towards which the opening inclined; the catheter remaining in the urinary passage. A bladder was at first attached to this instrument, but, being found inconvenient, the latter was secured by a T bandage, and the urine permitted to escape on a blanket. In the course of the night the catheter slipped from the passage, and was not replaced till the next morning. The patient, also, tired of the position in which she was left, became restless, and moved about in all directions. Notwithstanding these adverse circumstances, the urine passed only through the natural channel.

When I visited the patient on the fourth day, I found the catheter had again escaped from the urethra, and had not been replaced during the last two nights. Having laid her on the table in the same position as before, and having expanded the vagina, and introduced the female catheter into the urethra, I depressed the bladder, and divided the ligatures with a probe-pointed bistoury and a director bent towards the point. The threads were then removed with a pair of common forceps; and, from the view we could obtain, the union appeared to be complete. In order, however, to be satisfied respecting the cure, I left the catheter in the bladder during a quarter of an hour, having previously secured the lower opening with a plug. At the end of this time the plug was removed, and I had the pleasure of seeing the urine flow in a stream, while the vagina remained quite free from that fluid. In order to promote the retentive power of the bladder, I desired that the catheter might remain in the passage, and that the plug may be removed at stated intervals. The woman was also allowed to sit up, walk, or lie down, as she might feel disposed.

On the eighth day I examined the cicatrix again, and found, by passing the catheter in the urethra and the finger in the vagina, that no vestige of the fistula remained. As the woman had been very impatient, and had retained the instrument only occasionally in the passage, I desired that it might be discontinued.

On the sixteenth day she rode to Bridgnorth, a distance of seven miles from her residence. At that period she had so far regained the power of retaining the urine, that she could pass three ounces at a time. It now and then escaped involuntarily, particularly during the night; and she did not recover the retentive faculty of the bladder until the end of five months after the operation.

*Lancet. October* 6, 1838.

---

*Case of Strangulated Mesenteric Hernia.* By R. Ranking, Esq., Hastings.

[This is one of those cases of internal strangulation, so generally fatal, which are so well and fully described by Rokitanski, in our third Volume, p. 495. The symptoms in Mr. Ranking's case were those of hernia. The following is the account of the appearances found on dissection:]

I found that the mesentery had been separated from its attachment to the under surface of the bowel for the space of an inch and a half. The loop thus formed was further diminished by the intestine taking a turn upon itself, so as to convert the loop into a figure of 8. Through one of these smaller loops a knuckle of intestines, six inches long, had passed, and become tightly strangulated. There were thus two distinct points of strangulation; one formed by the twisting of the intestine upon itself, the other by the passage of the knuckle of intestine through the loop. Both strangulations were so perfect that air could not be made to traverse them without great difficulty. The edges of this laceration of the mesentery were smooth, and did not exhibit any trace of effusion of blood. The intestines below the stricture were closely contracted and empty.

*Med. Gazette. November* 3, 1838.

---

*Simple Mode of dilating the Urethra.* By A. L. Wigan, Esq., Brighton.

Being called to a little boy, about seven years of age, labouring under suppression of urine, I found him in a state of agony and spasm really alarming. On examination, I perceived in the urethra, close to the pubes, a stone about the size of a small French-bean. The efforts to force it onwards were most violent, and likely to lead to rupture of the urethra, if not soon relieved. I thought of letting out the stone by an incision, but determined to try first what could be done by dilatation. It was clear that a bougie of sufficient size could not penetrate near enough to the stone to give relief: I therefore inserted the point of a syringe of such a size as could be commanded with one hand, and forced warm water into the anterior portion of the urethra with a steady pressure, and had the satisfaction of producing, in about ten or fifteen minutes, sufficient dilatation to allow the passage of the stone.

*Med. Gazette. October* 20, 1838.

